# Sustainable Strategies to Counteract Mycotoxins Contamination and Cowpea Weevil in Chickpea Seeds during Post-Harvest

**DOI:** 10.3390/toxins15010061

**Published:** 2023-01-11

**Authors:** Claudia Pisuttu, Samuele Risoli, Lorenzo Moncini, Cristina Nali, Elisa Pellegrini, Sabrina Sarrocco

**Affiliations:** 1 Department of Agriculture, Food and Environment, University of Pisa, Via del Borghetto 80, 56124 Pisa, Italy; 2 University School for Advanced Studies IUSS, Piazza della Vittoria 15, 27100 Pavia, Italy; 3 Biotechnical Instruments in Agriculture and Forestry Research Centre (CRISBA), ISIS “Leopoldo II di Lorena”, Cittadella dello Studente, 58100 Grosseto, Italy; 4 Nutrafood Research Center, University of Pisa, Via del Borghetto 50, 56124 Pisa, Italy

**Keywords:** *Cicer arietinum* L., mycotoxigenic fungi, mycotoxin occurrence, pest attack, nitrogen-controlled atmosphere, ozone

## Abstract

Mycotoxins contamination and pest infestation of foods and feeds represent a pivotal threat for food safety and security worldwide, with crucial implications for human and animal health. Controlled atmosphere could be a sustainable strategy to reduce mycotoxins content and counteract the vitality of deleterious organisms in foodstuff. Ozone treatment (O_3_, 500 ppb for 30, 60 or 90 min) and high nitrogen concentration (N_2_, 99% for 21 consecutive days) were tested in the post-harvest management of four batches of *Cicer arietinum* grains to control the presence of mycotoxigenic fungi and their secondary metabolites, as well as pest (i.e., *Callosobruchus maculatus*) infestation. At the end of the treatment, O_3_ significantly decreased the incidence of *Penicillium* spp. (by an average of −50%, independently to the time of exposure) and reduced the patulin and aflatoxins content after 30 min (−85 and −100%, respectively). High N_2_ concentrations remarkably reduced mycotoxins contamination (by an average of −94%) and induced pest mortality (at 100% after 5 days of exposure). These results confirm the promising potential of O_3_ and N_2_ in post-harvest conservation strategies, leading to further investigations to evaluate the effects on the qualitative characteristics of grains.

## 1. Introduction

The increasing occurrence of food/feed contaminants worldwide poses a huge threat to human and animal health. One of the major contaminants are mycotoxins, which annually cause enormous economic losses in the food industry and animal husbandry [1,2]. These low molecular weight metabolites produced by filamentous fungi (belonging to the phylum Ascomycota) contaminate various categories of foods and feeds [3]. Two groups of mycotoxigenic fungi exist: field fungi (such as *Fusarium* and *Aspergillus* spp.) that infect crops before harvest, and storage fungi (such as *Penicillium* spp.), which only occur after harvest [4,5]. According to a recent world survey based on around 97,000 analyses performed between January and December 2020 on more than 21,000 finished feed and raw commodity sources collected from 79 countries, the most prevalent mycotoxins were those produced by *Fusarium*, affecting more than the 60% of tested samples [Biomin, Inzersdorf-Getzersdorf, Austria, https://www.biomin.net/science-hub/world-mycotoxin-survey-impact-2021/, accessed on 4 January 2023], In 2020, Mesterhazy et al. highlighted how toxins are responsible for a loss of almost 700 mt during the harvest and storage of grains [6]. At any stage of the food production process (in the field, during harvest, during drying and transport, as well as during storage), the fungal production of mycotoxins can occur by exposing consumers to the risk of contamination, either directly through food consumption or indirectly through feed [7]. The most important mycotoxins are aflatoxins [mainly represented by aflatoxin B1 (AFB1), B2 (AFB2), G1 (AFG1), G2 (AFG2) and M1 (AFM1)], ochratoxins, fumonisins, trichothecenes, zearalenone, the emerging *Fusarium* mycotoxins, ergot alkaloids, *Alternaria* toxins, and patulin [8]. Of the approximately 400 compounds identified as mycotoxins, 30 have received significant consideration due to their harmful effects on both human and animal health (including genotoxicity and endocrine disruption [9]). Despite a huge number of published papers reporting the occurrence of mycotoxins on cereals and cereal-derived food products, in 2017, an analysis of 104 papers—from 2006 to 2016—was carried out, summarizing that mycotoxins are ubiquitously present in cereals and cereal-derived food products throughout the world [10]. If Africa and Asia showed the highest incidence (%) of cereals contaminated by aflatoxins and ochratoxins, respectively, South and North America registered the highest level of fumonisins and Europe the highest percentage of deoxynivalenol (trichothecenes)/zearalenone contamination [10].

Different physical, chemical and biological factors affect fungal colonization and mycotoxins production. Physical factors include environmental conditions such as temperature, relative humidity, pH, water activity, nutrients, insect infestation and other associated factors, which at specific rates are known to favor the growth of many types of fungi and the production of mycotoxins [2]. Biological factors are mainly related to the interactions between the colonizing toxic fungal species and the host, thus including features such as fungal species, strain specificity, levels of inoculation, the nature of the substrate, strain variation, the instability of fungal toxic properties, and insect damage [11].

Regulatory agencies have established strict legislative thresholds in order to keep the levels of mycotoxins in food/feed commodities under control. These limits range from below one to thousands of μg kg^−1^, depending on the (i) mycotoxin, (ii) type of product and (iii) country considered [12,13]. Consequently, there is an urgent need to develop a feasible and highly sensitive analytical method for mycotoxins detection [14] and reduce the contamination of mycotoxins in food/feed, in order to protect/preserve their quality and safety. Overall, the control of mycotoxin contamination follows two strategies: the prevention of their production (i.e., microbial inactivation) and detoxification (e.g., mycotoxin degradation [3]). In pre-harvest, the control of mycotoxins is based on control of the contamination levels in crops to be used as food/feed components. Generally, these systems are based on preventive strategies (such as the use of resistant varieties, crop rotation, tillage and the management of irrigation) which aim to avoid the development of contamination, operating on the predisposing factors that facilitate the production of mycotoxins. Although pre-harvest approaches should be preferred, in the perspective of preventing mycotoxin contamination, the development of toxic fungi is inevitable under certain environmental conditions [15]. Therefore, appropriate storage practices and other post-harvest control systems (at the microbiological, physical and chemical level) are necessary to minimize the final mycotoxin content of foods/feeds [16]. These traditional methods for the elimination/inactivation of mycotoxins have some limitations regarding (potential) safety issues, loss in the nutritional value and the palatability of feeds, cost implication and limited efficacy [17]. In recent decades, various detoxification approaches have demonstrated to be (i) highly effective in degrading mycotoxins into less toxic products, (ii) economically favorable and (iii) not environmentally harmful [18]. Among these, cold plasma—containing reactive oxygen and nitrogen species and free radicals—has received attention in recent years for use on cereals during storage, due to its lethal effect on microorganisms and its potential to decontaminate surfaces and improve shelf-life [19]. Nevertheless, their practical application in food/feed matrices is limited, since the degradation process under conditions of large-scale production is much more complex, and the experiments at lab-scale might not always reflect practices in industrial processing [1]. Possible reasons for this are that the degradation process can be easily affected by multiple factors such as temperature, relative humidity, pH, water activity, nutrients, insect infestation and types of contamination [2]. The relevance of studying naturally contaminated samples is consistent with the actual distribution conditions of mycotoxins in the field and/or in grains.

Since gas composition is considered one of the most important abiotic conditions that impacts fungal and pest growth, ozonation (i.e., the application of gaseous ozone, O_3_) is a simple technology for controlling insects and reducing mycotoxins in stored products, which does not leave harmful residues after application. Being unstable, O_3_ quickly degrades into oxygen (and related cytotoxic radicals) in a short period, oxidizing the vital cellular components (such as unsaturated lipids and proteins) of pathogenic microbes and storage pests by causing lysis and rapid cell death [20]. Consequently, O_3_ can inhibit fungal growth, sporulation and germination by offering a negligible loss of nutrients or sensory qualities in food/feed [21], making it a suitable candidate as a residue-free fumigant. For this reason, the application of O_3_ in food chains has been considered safe and effective by the WHO and is now recognized as a “green technology” for the fumigation of grains, fruits and vegetables [22]. In fact, O_3_-treated products are safe for consumption and their microbiological shelf life can be greatly enhanced. However, the efficacy of O_3_ in fungi count reductions, mycotoxin degradation and insect control depend on the (i) method, concentration and timing of the O_3_ application; (ii) microorganisms/contaminants to inactivate; (iii) the type and mass of food/feed processed; and (iv) other co-factors such as temperature, relative humidity and water activity [16,20]. Similarly, the use of a controlled or modified atmosphere by using a very high nitrogen (N_2_) concentration is a valid alternative to chemical fumigation to control mycotoxigenic fungi contamination and pest challenge post-harvest [23]. Its effects on different stored products (such as wheat, maize, corn and rye) are well documented [2,24]. In particular, a N_2_-controlled atmosphere can control fungal growth and proliferation by improving the quality of stored products [25]. The action of N_2_ at high concentration is mainly due to the significant reduction in O_2_ (1% or less [26]) and offers several advantages at the economic and environmental level [27]. A major advantage of N_2_ is that all gas is free of pollutants, leaving no residue in food/feed. Consequently, N_2_-treated products are safe for consumption and their microbiological shelf-life can be greatly enhanced [28]. Consequently, a N_2_-controlled atmosphere might represent an eco-friendly tool that could be transferred to a large-scale system for grain storage as an alternative strategy to the use of conventional residue-producing chemical fumigants [29]. However, the efficacy of a N_2_-controlled atmosphere in fungi count reductions, mycotoxin degradation and insect control depends on the (i) concentration of gas, (ii) the timing of the application, (iii) microorganisms/contaminants to inactive, (iv) the type and mass of food/feed processed, and (v) other co-factors such as temperature and water activity [30].

The chickpea (*Cicer arietinum* L.) is a legume of the family Fabaceae, subfamily Faboideae. It is one of the most cultivated pulses in terms of world production due to its low content of fat and sodium, absence of cholesterol and being an excellent source of both soluble and insoluble fiber, complex carbohydrates, vitamins, folate and minerals (such as calcium, phosphorus, iron and magnesium [31]). With a worldwide production of more than 12 million tons per year [FAOSTAT, https://www.atlasbig.com/en-in/countries-by-chickpea-production, accessed on 4 January 2023], chickpea represents one of the five leading pulses based on sales value. In 2021, India was the largest chickpea producer in the world, with around 11 million metric tons of production, followed by Turkey, with around 600,000 metric tons (https://www.statista.com/statistics/722203/chickpeas-production-volume-by-country-worldwide/, accessed on 4 January 2023). Chickpea is often attacked by fungi pre- and post-harvest, significantly affecting its productivity. Many fungal genera/species commonly isolated from chickpea seeds and chickpea by-products are potential mycotoxin-producers, especially of aflatoxins, ochratoxin A and patulin, so there would be a potential risk of contamination [32]. Another issue threatening chickpea quality is *Callosobruchus maculatus* (Fab.) (Coleoptera: Chrysomelidae: Bruchinae [33]), which is also known as the “cowpea weevil”. The granivorous larvae of cowpea weevil are the considerable causative agent of severe losses in the grain germination, weight and nutritional values of chickpea (in some cases reaching 60% of the grain [34]). In addition, *C. maculatus* can favor the occurrence of infections due to mycotoxigenic fungi (*Aspergillus* and *Penicillium* [35]).

**Figure 1:**
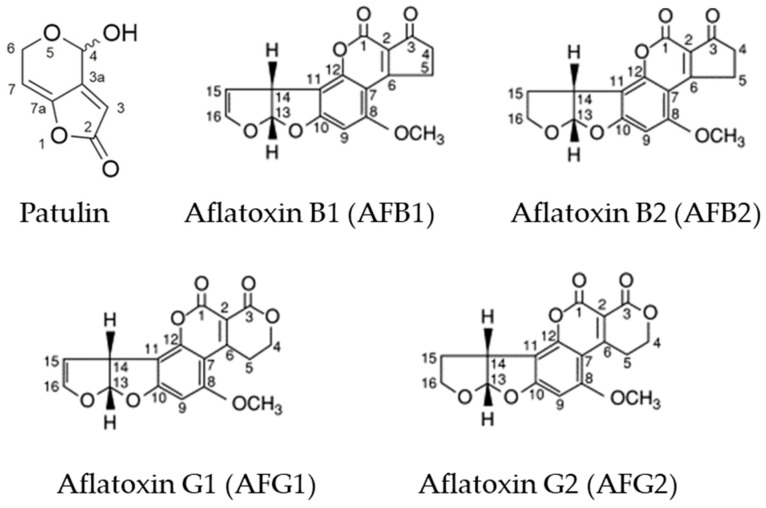
Molecular structures of measured mycotoxins (patulin, aflatoxin B1, aflatoxin B2, aflatoxin G1 and aflatoxin G2; modified by [3]), showing numbering of carbon atoms (3a and 7a represent small fragments).

The aim of this work was to investigate the possibility of using a single pulse of O_3_ or high N_2_ concentrations as storage technologies (at lab- and large-scale) for the purpose of (i) containing the fungal population present on the chickpea seeds surface; (ii) reducing the mycotoxins content (such as aflatoxins and patulin; Figure 1); and (iii) limiting the *C. maculatus* infestations (only in the case of N_2_ treatment). We postulated that O_3_ and N_2_ can be an alternative to traditional chemical-based fumigants for controlling spoilage pathogens and insects in stored chickpea seeds.

## 2. Results

### 2.1. Effect of O_3_ and N_2_ Treatments on Fungal Infection

To evaluate the effect of O_3_ treatment as well as of the conservation under a N_2_-controlled atmosphere on a fungal population naturally occurring on chickpea seeds, with particular attention to mycotoxigenic fungi, a grain health test was performed on control stocks stored at 4 °C and on seeds after O_3_ or N_2_ treatments. After the morphological and molecular identification of single colonies isolated from seeds, all the batches were contaminated with *Penicillium* spp. isolates, but at a different extent depending on the quality of the batches. Molecular identification performed on the ITS region sequence confirmed the membership of all the isolates to this important fungal genus, with the species *P. pinophilum* and *P. polonicum* as the most represented.

**Figure 2:**
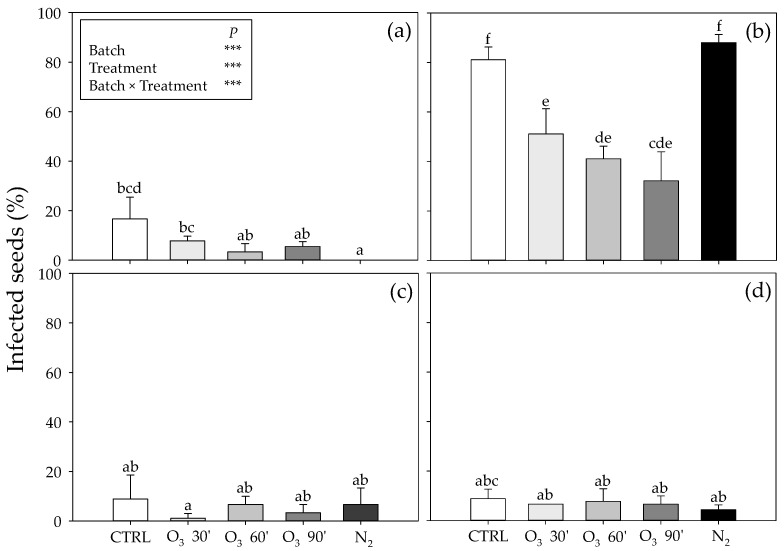
Contamination levels (% of infected seeds) by *Penicillium* spp. in four batches (n. 1 (**a**), n. 2 (**b**), n. 3 (**c**) and n. 4 (**d**)) of chickpea seeds (CTRL, white fill) exposed to ozone [500 ppb O_3_ for 30 (O_3_ 30’, light grey fill), 60 (O_3_ 60’, grey fill) and 90 (O_3_ 90’,dark grey fill) minutes] or nitrogen treatment (99% N_2_ for 21 consecutive days, dark fill). Data are shown as mean + standard deviation (n = 3). Results of two-way ANOVA are reported; asterisks show the significance of factors/interaction for: *** *p* ≤ 0.001. According to Tukey’s HSD post hoc test, different letters indicate significant differences (*p* ≤ 0.05).

Of particular interest was batch n. 2, where almost 80% of the analyzed seeds were infected by *Penicillium* isolates, thus, confirming the non-marketability of this batch for commercial purposes. The other three batches showed a percentage of natural occurring infection varying around 10–20% (Figure 2).

After O_3_-exposure, as well as after incubation under the N_2_-controlled atmosphere, the grain health test, in most cases, resulted in a reduction in the *Penicillium* contamination of the seeds. A significant effect of single factors and their interaction was observed. For batch n. 1, O_3_ treatment did not result in any significant reduction if compared to the control, as well as among treatments. Conversely, a significant reduction was registered after N_2_ incubation (−16% of infected grains compared with no seed developing *Penicillium* spp. colonies; Figure 2a). Much more evident was the effect of the O_3_ treatment on batch n. 2, where, independently from the time of exposure, a significant reduction in the naturally occurring *Penicillium* population was observed (−55% as average), with the exposure lasting 90 min being the most efficient (−75%). On the other hand, any significant difference was observed after the incubation in the N_2_-controlled atmosphere (Figure 2b). For batches n. 3 and 4, a similar behavior was observed, where any significant difference in the percentages of infected seeds was observed if compared with the controls, as well as among treatments (Figure 2c,d). However, in these last two cases, the initial infection was lower if compared with the other two batches, particularly with batch n. 2.

### 2.2. Effects of O_3_ and N_2_ Treatments on Mycotoxin Levels

**Figure 3:**
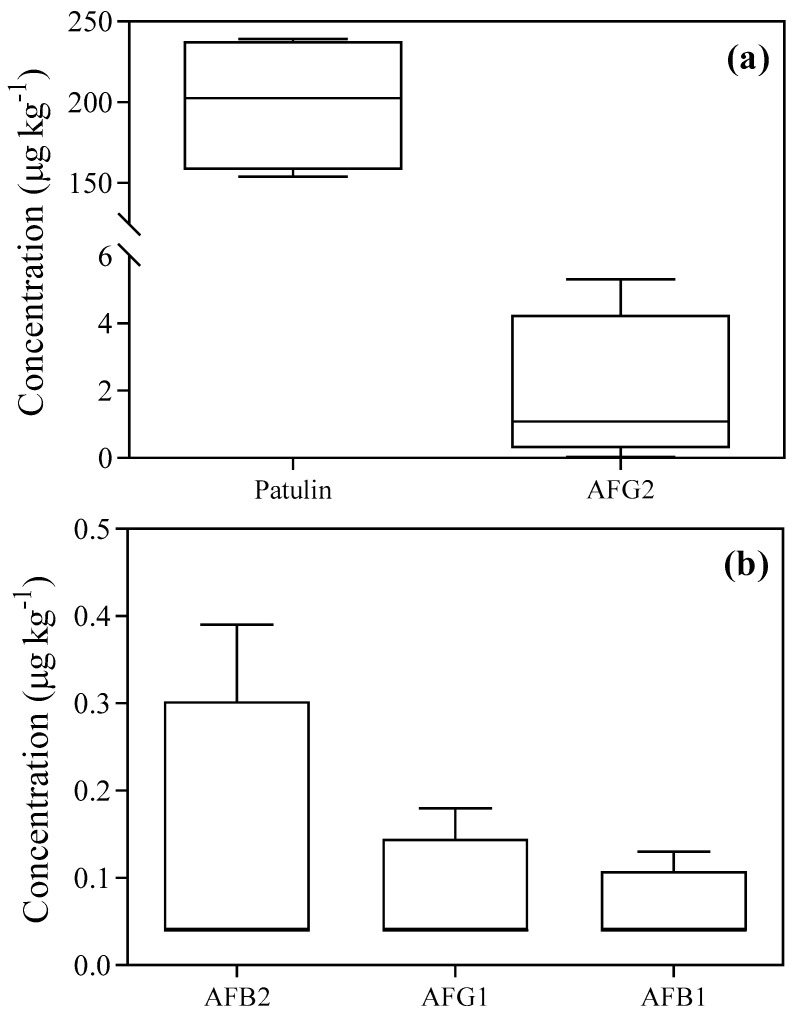
Box and whiskers representation of the average content of measured mycotoxins in four batches of chickpea seeds ((**a**) patulin and aflatoxin G2 (AFG2); (**b**) aflatoxin B2 (AFB2), aflatoxin G1 (AFG1) and aflatoxin B1 (AFB1)). For each mycotoxin, the top line represents the 90^th^ percentile; the bottom line represents the 10^th^ percentile; and the box represents the 75^th^ percentile (upper side), the 25^th^ percentile (lower side) and the median (50^th^ percentile, central line), respectively.

In the four batches of chickpea grains maintained in filtered air or incubated in control silos, the mycotoxins patulin, AFG2, AFB2, AFG1 and AFB1 were found. Figure 3 shows an overview of the concentrations of these mycotoxins and their variability among the batches.

The comparison between the content of selected mycotoxins and the batches of chickpea grains maintained in filtered air or incubated in control silos revealed that the concentrations of patulin were significantly higher in batch n. 4 than those in batch n. 1 (+55%; Table S1). No significant differences were observed among the remaining batches of chickpea grains. The levels of AFB1, AFB2 and AFG1 were significantly higher in batch n. 3 than those in the remaining batches of chickpea grains (about six-fold higher on average). Conversely, the levels of AFG2 were significantly higher in batch n. 2 than those in the remaining batches of chickpea grains (about 100-fold higher than batch n. 1, and five-fold higher than batches n. 2 and 3; Table S1).

**Figure 4:**
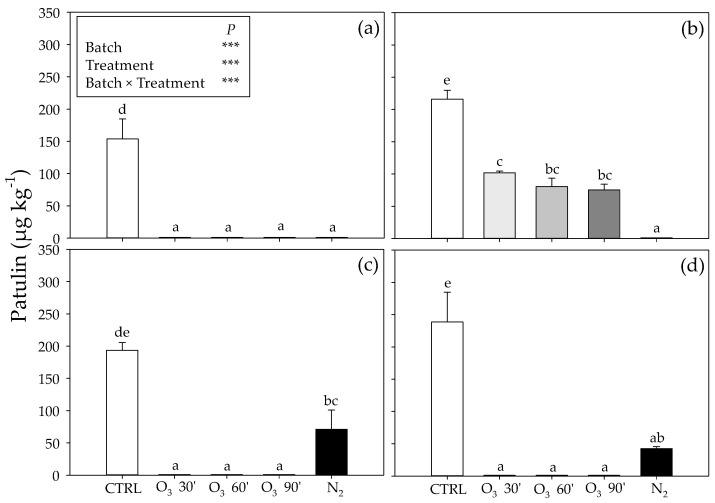
Patulin content in four batches (n. 1 (**a**), n. 2 (**b**), n. 3 (**c**) and n. 4 (**d**)) of chickpea seeds (CTRL, white fill) exposed to ozone (500 ppb O_3_ for 30 [O_3_ 30’, light grey fill), 60 (O_3_ 60’, grey fill) and 90 (O_3_ 90’, dark grey fill) minutes] or nitrogen treatment (99% N_2_ for 21 consecutive days, dark fill). Data are shown as mean + standard deviation (n = 3). Results of two-way ANOVA are reported; asterisks show the significance of factors/interaction for: *** *p* ≤ 0.001. According to Tukey’s HSD post hoc test, different letters indicate significant differences (*p* ≤ 0.05).

The effects of the O_3_ and N_2_ treatments on the patulin levels in the four batches’ grains are reported in Figure 4. The two-way ANOVA test revealed that the interaction “batch of chickpea grains × treatment” and the effects of each factor were significant. Ozone treatment induced a significant decrease in patulin in batch n. 2, independently of its duration (an average of −50% compared to chickpea grains maintained in filtered air; Figure 4b), and even more in the remaining batches (about 150-, 230- and 240-fold lower than CTRL in batches n. 1, 3 and 4, respectively). Similarly, a reduction in patulin was observed in batches n. 3 and 4 incubated in silos under high N_2_ concentrations (−70 and −82% than CTRL, respectively; Figure 4c,d), and even more in the remaining batches (about 160-fold lower than those in batches n. 1 and 2 incubated in control silos, respectively).

The effects of the O_3_ and N_2_ treatments on the total aflatoxin levels in four batches of chickpea grains are reported in Figure S1. The two-way ANOVA test revealed that the interaction “batch of chickpea grains × treatment” and the effects of each factor were significant. Ozone treatment induced a complete reduction in total aflatoxins in batches n. 2, 3 and 4, independently of its duration (about 80-fold lower compared to chickpea grains maintained in filtered air). Similarly, a reduction in total aflatoxins was observed in batches n. 2, 3 and 4 incubated in silos under high N_2_ concentrations (about 150-fold on average). No other significant differences were found in batch n. 1, independently of the kind of treatment.

### 2.3. Effect of N_2_ Treatment on Pest Survival

**Figure 5:**
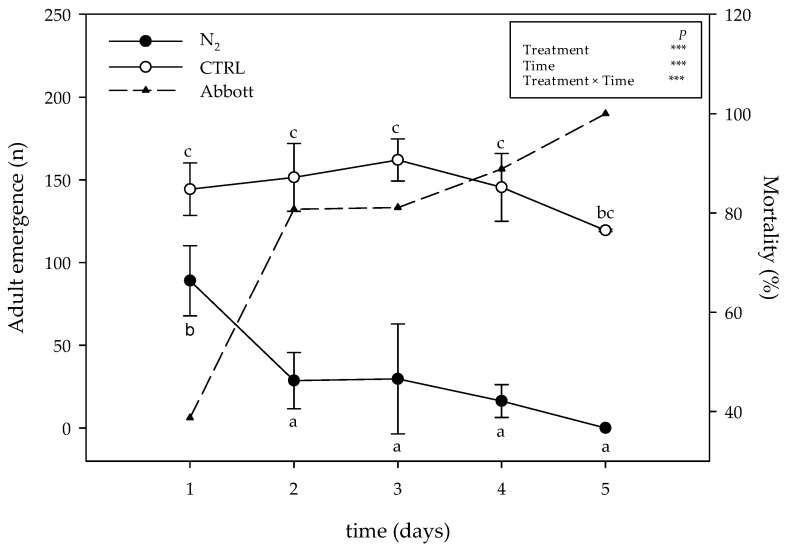
Number of emerged adults of *Callosobruchus maculatus* and mortality (measured using Abbott’s index, dotted line and dark triangle) on batch n. 1 (CTRL, open circle) and exposed to nitrogen treatment (60 L lab-scale; dark circle). Data are shown as mean ± standard deviation (n = 3). Results of two-way ANOVA are reported; asterisks show the significance of factors/interaction for: *** *p* ≤ 0.001. According to Tukey’s HSD post hoc test, different letters indicate significant differences (*p* ≤ 0.05).

The effect of 5-day exposure in the N_2_-controlled atmosphere (in the 60 L lab-scale silos) on the number of emerged adults and on Abbott’s index is reported in Figure 5.

A statistical analysis highlighted how not only the treatment and the time had a significant effect on the adult emergence, but also the interaction between these two sources of variability resulted as highly significant (*p* ≤ 0.001). The exposure to the N_2_-controlled atmosphere resulted in a reduction in the number of emerged adults from the first day of exposure. No significant differences were observed after 24 h of exposure. From the second until the fifth day, a continuous and significant reduction was observed, with the adult emergence close to zero after five days of exposure of the eggs within the silos under the N_2_-controlled atmosphere.

With respect to Abbott’s index, which is conventionally used to evaluate the effect of treatments on pest mortality compared to naturally occurring mortality, the incubation of eggs under high N_2_ concentration resulted in an increasing mortality over time of exposure, with 100% reached at the end of the experiment, i.e., after 5 days of exposure (Figure 5, dashed line).

**Figure 6:**
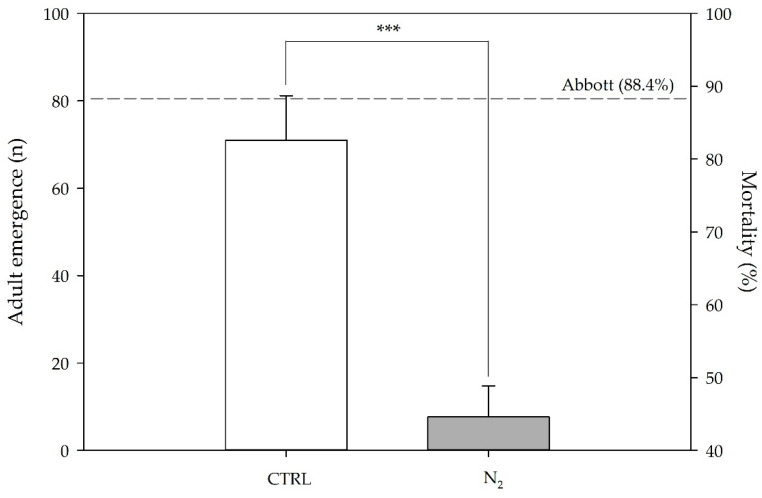
Number of emerged adults of *Callosobruchus maculatus* (Fab.) and mortality (measured using Abbott’s index, dotted line) on batch n. 1 (CTRL, white fill) and exposed to nitrogen (N_2_) treatment (real-scale; gray fill). Statistical differences were examined by paired Student’s *t*-test; asterisks indicate statistical significance for: *** = *p* ≤ 0.001.

When the effect of the high N_2_ atmosphere was evaluated on *C. maculatus* adult number in real-scale silos after 5 days of exposure (Figure 6), a highly significant reduction in emergence was registered, compared to the control. This result was also confirmed by Abbott’s index, which resulted in approximately 80% of mortality due to the N_2_-controlled atmosphere.

## 3. Discussion

In the present study, the fungicidal efficacy of O_3_ was only observed in batch n. 2 (the most infected seeds), as confirmed by the large reduction in *Penicillium* contamination. According to Mendez et al. [36], at the beginning of O_3_ treatment, this gas reacts with a mass of grains and quickly decomposes. In its second phase, O_3_ moves freely through the grains with little degradation. Moreover, O_3_ reacts faster with the whole mass of grains when higher dosages are used. This was also confirmed by our results: a higher reduction in the naturally occurring fungal population was observed by using O_3_ at 500 ppb for 90 min. To date, few studies have investigated the direct effect of O_3_ on fungal growth and proliferation, preferring to focus on the reduction in mycotoxins in different products (such as wheat, corn, corn flour, peanuts and pistachio [37]). Most studies have been carried out on aflatoxins. Using O_3_ in patulin, degradation has only been studied in apple juice, apples and pear with brown rot, flour, and malt feed [38]. In the present study, O_3_ treatment induced a significant decrease in patulin in batch n. 2 (independently of its duration), and even more in the remaining batches. The mechanism of patulin degradation might be associated with the oxidation of a polyketide lactone on its structure, which made it highly susceptible to O_3_ attack [39]. The experimental conditions used in this study (e.g., O_3_ and/or the timing of treatment) were sufficient, by attacking two conjugated ethylenic double bonds on the chemical structure of patulin and inducing its partial or full degradation [40]. Similarly, all aflatoxins were easily attacked and degraded by O_3_ (independently of its duration), confirming its efficacy as a detoxifying agent. The mechanism of AFB1 and AFG1 degradation might be associated with the oxidation of the C8-C9 double bond at the terminal furan, resulting in the production of primary ozonide [26]. This product may rapidly rearrange to a molozonide derivative, yielding a variety of carbonyl compounds or organic acids. Since AFB2 and AFG2 lack a susceptible double bond for oxidation, their degradation requires higher levels of O_3_ and/or longer exposure until the lactone ring is opened [41]. The experimental conditions used in this study (e.g., O_3_ concentration and/or the timing of treatment) were sufficient, by rapidly and effectively detoxifying aflatoxins without any difference in degradation rate between AFB1 and AFG1 with AFB2 and AFG2 (the most abundant aflatoxin in batches n. 2, 3 and 4). It is worth noting that the production of aflatoxins was not associated with the presence of the fungal itself, confirming that the absence of *Aspergillus* spp. from chickpea seeds does not guarantee the absence of aflatoxins because of their resistant chemical nature [42].

In the present study, high N_2_ concentrations only induced a reduction in *Penicillium* contamination in batch n. 1 confirming that certain fungal species might continue to grow, albeit at a greatly reduced rate under low O_2_ concentrations [43]. However, a significant decrease in patulin was observed in batches n. 3 and 4, and even more in the remaining ones, indicating that low O_2_ concentrations may (partially or totally) depress patulin production by *Penicillium* spp. on chickpea seeds. In addition, the experimental conditions used in this study (e.g., N_2_ concentration and/or the timing of treatment, temperature, relative humidity and water activity) were sufficient, by totally removing aflatoxins. Consequently, it is possible to speculate that high N_2_ concentrations are more effective in inhibiting selected mycotoxins (aflatoxins > patulin) than in preventing the development of mycotoxigenic fungi [44]. Although a controlled atmosphere is used to control both mycotoxigenic fungi and insects in stored products, it has been documented that the experimental conditions sufficient for controlling fungal proliferation are not always effective against insect pests that can survive, due to the dependence on other environmental factors (e.g., temperature and humidity [45]). This is in line with the results of the present work, where the same experimental conditions (e.g., N_2_ concentration and/or the timing of treatment, temperature, relative humidity and water activity) that were partially effective for reducing the growth of *Penicillium* spp. were effective for detoxifying mycotoxins (as previously reported) and limiting *C. maculatus* infestation (as confirmed by the reduction in adult emergence already observed starting from the first day of the experiment, and the concomitant pest mortality). This is of relevance, since it is generally recognized that pest attack can damage grains and favor moisture accumulation by creating suitable conditions for fungal development and mycotoxin production (as observed in batch n. 2 [46]). Consequently, it is possible to speculate that the experimental conditions used in this study were sufficient, by limiting the pest infestation and guaranteeing seed quality, as already reported in wheat [23,25]. In addition, the possibility of moving to a real-scale dimension, as here preliminary reported, with results comparable with those obtained in the 60 L lab-scale silos, marks a further step made in the direction of the scaling-up of the method. In fact, the set-up chosen for the present study is not only a suitable way to provide a proof-of-concept of its efficacy before scale-up, but also a valid choice for small and medium farms [23,25].

**Figure 7:**
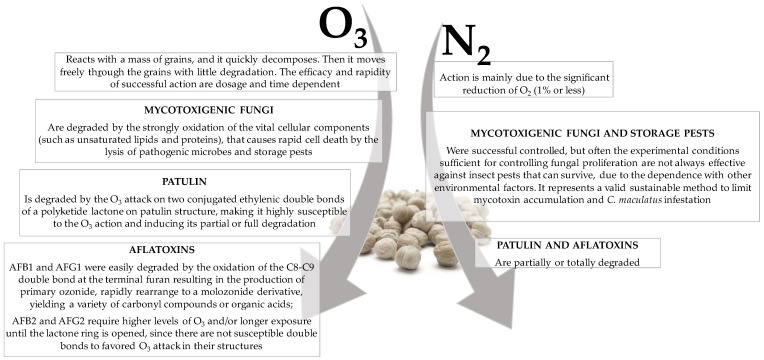
Gaseous ozone (O_3_) and nitrogen (N_2_) mechanisms of action during post-harvest of grains, and their effects on (i) mycotoxigenic fungi and storage pests (only N_2_), (ii) patulin and (iii) aflatoxins.

The findings of the present study are relevant in enhancing the shelf-life of chickpea seeds by controlling fungal growth, mycotoxin contamination and pest infestation with eco-friendly and low-cost storage practices (the mechanisms of action of O_3_ and N_2_ are summarized in Figure 7).

In particular, O_3_ can contain the fungal population present on the chickpea seeds surface and reduce the content of patulin and aflatoxins. This is a fundamental goal in the development of emerging new techniques, since these metabolites are recognized as a Group I carcinogen by the International Agency of Research on Cancer (Lyon, France), and their allowable levels in human foods and animal feedstuff are strictly regulated by governmental jurisdictions in about 100 countries [47]. It is worth noting that the antimicrobial activity of O_3_ is highly dependent on vegetable/fungus species, growth stage, concentration and timing of exposure. Improvements and innovations in O_3_ generation and application systems will be evaluated more effectively in the future by facilitating the enhanced control of both the quality and safety parameters of ozonized foods/feeds. Similarly, a N_2_-controlled atmosphere represents a valid sustainable method to limit mycotoxin accumulation and *C. maculatus* infestation. This system requires low energetic costs, offers a negligible loss of nutrients or sensory qualities in food/feed, and demonstrates a reduced hazard to employees with no need for registration and no contamination of the environment [23,25]. Consequently, it can be considered as a promising alternative method that could be transferred to a large-scale grain storage system. For effective and safe use in processing, optimum O_3_ and N_2_ concentrations, contact time and other treatment conditions should be defined for foods and feeds. Here, a pilot test was conducted by offering scientific evidence to support the commercial application of these innovative strategies. 

## 4. Conclusions

In conclusion, our pioneering study demonstrated that the experimental conditions used (e.g., O_3_ and N_2_ concentrations and/or the timing of treatment) were enough to rapidly and effectively (i) reduce the growth and proliferation of *Penicillium* spp. population present on the chickpea seeds’ surface (in the case of O_3_ treatment); (ii) detoxify patulin and aflatoxins; and (iii) limit *C. maculatus* infestation (only in the case of N_2_ high concentrations). These are fundamental goals in the development of emerging new techniques and novel methods to control mycotoxin infections, intoxications and diseases, by offering a negligible loss of nutrients or sensory qualities in stored products and without leaving toxic chemical residues in the food and feed chain.

Therefore, our results suggest that a single pulse of O_3_ and high N_2_ concentrations are a promising alternative to traditional chemical-based fumigants for controlling spoilage pathogens and insects in stored chickpea seeds. An industrial facility of O_3_ technology remains to be developed for the large-scale treatment of food/feed products, requiring input from different disciplines. For effective and safe use in processing, optimum O_3_ and N_2_ concentrations, contact time and other treatment conditions (e.g., vegetable/fungus species) should be defined for foods and feeds. 

Additional research is obviously required to evaluate the responses of stored products to these effective and straightforward solutions, in order to control mycotoxin contamination and pest infestation at post-harvest. This would provide a clearer picture of the practical advantages of O_3_ and N_2_ treatment and allow further endorsement of our present results.

## 5. Materials and Methods

### 5.1. Reagents and Standards

Sodium hypochlorite, ethanol and streptomycin sulphate were supplied by Sigma-Aldrich (Milan, Italy). Potato Dextrose Agar was purchased from Biolife (Milan, Italy). Acetonitrile, methanol and water were HPLC-grade (Carlo Erba, Milan, Italy). Standards of patulin and aflatoxins were chromatographically pure and purchased from Sigma-Aldrich (Milan, Italy) and Romer Lab (Getzersdorf, Austria).

### 5.2. Raw Materials

Four *C. arietinum* batches, produced by a local farm located in Tuscany (Italy), were used in the present work. Before commercialization, all the batches (except in the case of n. 3) were submitted to a quality check by the producer, thus resulting in batches n. 1 and n. 4 being validated for sale, and batch n. 2 rendered non-compliant. All seeds were stored at 4 °C until submitted to a grain health test, mycotoxin determination and treatments under a controlled atmosphere.

### 5.3. Isolation and Identification of Fungal Contaminants Naturally Associated with Chickpea Seeds

To assess the presence of potential mycotoxigenic fungi naturally associated with chickpea seeds, all four batches were submitted to a grain health test. In detail, seeds from each batch were surface sterilized on a rotary shaker for 1 min in a solution containing NaClO (1% active chlorine) in 50% ethanol, then washed three times in sterile distilled water for 1 min each. After drying on filter paper, the seeds were transferred to 100 mm diameter Petri dishes containing Potato Dextrose Agar (PDA, 42 g L^−1^) with the addition of 300 mg L^−1^ of streptomycin sulphate. Since batch n. 2 showed a profuse development of *Mucor* spp., the seeds from this sample were plated on PDA that contained the antibiotic, as previously described, and with hymexazol fungicide (at the final concentration of 300 mg L^−1^; [48]). For each batch, four replicates (each consisting of twenty-five seeds) were made. The plates were incubated at room temperature (24 ± 2 °C). Then, from the second to the tenth day of incubation, colonies morphologically attributable to *Aspergillus* and *Penicillium* spp., developing from seeds, were transferred to new PDA + streptomycin sulphate plates and incubated under the same conditions previously reported. Then, when sporulated, they were used for single-spore cultivation. The single-spore *Penicillium* spp. colonies were used for molecular identification. Genomic DNA was extracted from each single-spore culture according to the Chelex 100 method [49]. For molecular identification, the complete internal transcribed spacers (ITS) 1 and 2 sequences—including the 5.8S gene—of the nuclear ribosomal DNA were amplified and sequenced as described in Sarrocco et al. [30]. All the sequences were then submitted to GeneBank (NCBI) to assign, where possible, the species for a preliminary evaluation of the risk of mycotoxin contamination that could occur on the seeds.

### 5.4. O_3_ and N_2_ Treatment Systems for Chickpea Seeds

#### 5.4.1. O_3_ Treatment System for Chickpea Seeds

Chickpea seeds (300 g from each batch) were placed in two Perspex chambers (60 × 60 × 110 cm) in a controlled environment fumigation facility. The system was adapted by including commercial colanders (34 × 23 cm, stainless steel), collocated in the middle of the chambers, in which seeds were placed to allow their complete exposure to O_3_, maintained in the dark throughout the whole period of the experiment (temperature 25 ± 1 °C, relative humidity (RH) 50 ± 5%). The fumigation system was continuously ventilated (two complete air changes per min) with charcoal-filtered air. Fumigation was performed by generating O_3_ from pure oxygen by electrical discharge, using a Fisher 500 air-cooled apparatus (Fisher America Inc., Houston, TX, USA). The O_3_ concentration was monitored with a Serinus 10 analyzer (Ecotech Acoem Group, Milan, Italy) set at 500 ± 50 ppb of O_3_ (for O_3_, 1 ppb = 1.96 μg m^−3^, at 20 °C and 101.325 kPa) for 30, 60 and 90 min, in which the chickpea seeds were mixed every 15 min. At the end of each treatment, the samples were collected and immediately used for the subsequent analyses (fungal infection and mycotoxin contamination). The entire methodology was performed according to Marchica et al. [50].

#### 5.4.2. Nitrogen Treatment System for Chickpea Seeds

Nitrogen treatments were performed using a NitrosepAgri system (Eurosider sas, Grosseto, Italy) based on selective membrane (MNS, Membrane Nitrogen Separator) to separate N_2_ from atmospheric air [25]. The N_2_-enriched atmosphere was driven into silos where it was maintained under a slight overpressure. All the environmental parameters, such as temperature and RH, were constantly monitored, and the N_2_ percentage could be set, automatically maintained and quickly reintegrated if needed. To perform the experiments here reported, two lab-scale (20 and 60 L silos, respectively) prototypes, already described in Moncini et al. [25], and one field-scale apparatus were used. For each chickpea batch, 300 g of seeds were transferred into a 1.5 L glass jar (9 cm diameter) that was closed with a micro-perforated nylon layer (350 μm pore size) to facilitate gas exchange and incubated under a 99% N_2_ atmosphere for 21 consecutive days. For each treatment and for each batch, three replicates were made. The field-scale apparatus consisted of four 15 m^3^ volume fiberglass silos connected to the MNS system. Each silos had a stainless steel (786 mm diameter) top hatch for grains charging, equipped with an overpressure valve and, on the bottom, with a stainless-steel ball (90 mm diameter) valve for discharging the product. The field-scale apparatus is located at the Azienda Agraria Macchiascandona (Castiglione della Pescaia, Grosseto, Italy).

### 5.5. Effect of O_3_ and N_2_ Treatments on Fungal Infection, Mycotoxin Contamination and Pest Survival

At the end of the O_3_ and N_2_ treatments, the seeds were collected and used to evaluate fungal contamination according to the grain health protocol. Mycotoxins determination was performed by using the clean-up aflatoxins and patulin (AFP) columns (OR SELL, S.p.a., Modena, Italy), according to the manufacturing protocols, with a few modifications. The samples were extracted by adding 50 mL of acetonitrile:water (ACN:H_2_O, 84:16 *v*/*v*) solution to 25 g of finely ground chickpea seeds, and vigorously vortexed for 6 min. The samples were centrifuged for 10 min at 12,000× *g* at room temperature, and the supernatants were filtered through Whatman^®^ paper (Cytiva, Marlborough, MA, USA) and subsequently by using the clean-up AFP columns, which enable the contemporary purification of aflatoxins and patulin. The obtained solutions were equally separated, dried at 40 °C and finally resuspended in 400 µL of 45% methanol (*v*/*v*) or 75% ACN (*v*/*v* in HPLC-demineralized water) for aflatoxins and patulin separation, respectively. The separation was performed in a UHPLC Dionex UltiMate 3000 system (Thermo Scientific, Waltham, MA, USA) equipped with a ZORBAX Eclipse Plus C18 column (150 × 4.6 mm, 5 μm particle size, Agilent technologies, Santa Clara, CA, USA). Aflatoxins determination was carried out by using an UltiMate™ 3000 Fluorescence Detector (Thermo Scientific, Waltham, MA, USA) with excitation and emission at 362 and 420 nm, after post-column derivatization through a UVE™ Photochemical Reactor for Aflatoxin Analysis (254 nm lamp; 240 VAC, 50/60 Hz, LCTech, Obertaufkirchen, Germany). The run conditions were set at a flow rate of 0.8 mL min^−1^ of a mobile phase 45% methanol (*v*/*v* in HPLC-demineralized water), for 30 min at 30 °C. Patulin quantification was performed using a Dionex UV-Vis Detector (Dionex UVD 170 U UV-Vis detector, Thermo scientific, Waltham, MA, USA) at 276 nm, at a flow rate of 0.6 mL min^−1^, for 30 min at 30 °C, and the same mobile phase reported above. Known amounts of pure (Patulin HPLC standard) or mixed (Aflatoxin Mix 4 solution) standards were injected into the UHPLC system (range 0.1−100 ng mL^−1^), and the quantity of each mycotoxin was obtained by correlating the peak area to the related standard concentration by using the Chromeleon Chromatography Management System software, version 7.2.10-2019 (Thermo Scientific, Waltham, MA, USA). The sum of AFB1, AFB2, AFG1 and AFG2 was considered as a measure of the total aflatoxins content.

Stock cultures of *C. maculatus*, kindly provided by Graham J. Holloway (University of Reading, Reading Berkshire, UK), were maintained in a climatic chamber (25 ± 1 °C, 70 ± 5% RH) on chickpea seeds in a 150 mL PP jar (5.5 cm diameter) that was closed with a micro-perforated nylon layer (350 μm pore size) to facilitate air exchange. To obtain chickpea infested with eggs, pest sub-cultures were set up by placing 100 unsexed adults into glass Petri dishes (14 cm diameter) containing 100 g of chickpea seeds. Adults were allowed to oviposit for 24 h in a climatic chamber under the same conditions as described before. At the end of the oviposition period, the adults were removed. Seeds with the addition of age-synchronized eggs were immediately used for the N_2_-controlled atmosphere test [51]. The lab-scale experiment was set up in six 60 L silos (three under a 99% N_2_ atmosphere and three under an unmodified atmosphere used as control). Two bio-tests, each consisting of 15 g of chickpea seeds with the addition of 1-3 eggs (up to a total of 30 eggs) placed into a 30 mL PP jar (4.3 cm diameter) and covered with a micro-perforated nylon layer (350 μm pore size), were transferred into each of the 60 L silos, which were already full of chickpea seeds; one bio-test was placed at the center and the other 10 cm under the grains’ surface. The infected seeds were incubated for 24, 48, 72, 96 and 120 days. To avoid the opening of the silos at each sampling, the experiment was repeated each time, with three replicates for each experiment.

With respect to the field-scale experiment, two silos filled with 9 tons of chickpea seeds (the same batch as the laboratory) were used for the tests: one of these was automatically kept at a 99% N_2_-controlled atmosphere, and the other one, with the lid partially opened and disconnected from the N_2_ supply, was used as a control. In order to simulate a storage condition inside the full grain mass, three bio-tests previously placed into a jute bag filled with 8 kg of chickpea seeds were transferred into the silos. The test was carried out for 5 days and replicated three times. At the end of each experiment (lab-scale and large-scale test), the bio-tests were removed from the silos and their contents transferred into larger PP jars (like those used for pest stock cultures), closed with a micro-perforated nylon layer and placed in a climate chamber at 25 ± 2 °C, 70 ± 5% RH. The adults’ emergence was periodically recorded from 35 to 70 days after oviposition. The number of adults was registered, and mortality was corrected using Abbott’s formula [52].

Mortality (%) = [(N_c_ − N_t_)/(N_c_)] × 100

where N_c_ = No. of emerged adults in control and N_t_ = No. of emerged adults in treatment.

### 5.6. Statistical Analysis

The robustness of data among the replicates was verified according to the results of the Shapiro–Wilk test for normality and Levene’s tests for homogeneity of variance. Data were submitted to an analysis of variance (one-way or two-way ANOVA), and comparisons among the means were determined by Tukey’s HSD post hoc test or Student *t*-test by using JMP Pro 14 software (SAS Institute Inc., Cary, NC, USA), in order to evaluate the effect of the treatments (control silos; O_3_ exposure for 30, 60 and 90 min; and N_2_ exposure for 21 consecutive days), batch (B1-4), and their interaction. For all the analyses, *p* ≤ 0.05 was assumed as a significant level.

## Data Availability

Not applicable.

## Supplementary Materials

The following supporting information can be downloaded at: https://www.mdpi.com/article/10.3390/toxins15010061/s1, Table S1: Analysis of variance of mycotoxins content in the four chickpea batches between the mean values determined on three different replicates; Figure S1: Total content of aflatoxins in four batches [n. 1 (a), n. 2 (b), n. 3 (c) and n. 4 (d)] of chickpea grains (CTRL, white fill), exposed to ozone [500 ppb O_3_ for 30 (light grey fill), 60 (grey fill) and 90 (dark grey fill) minutes] and nitrogen treatment [99% N_2_ for 21 consecutive days, dark fill]. Results of two-way ANOVA are reported, asterisks show the significance of factors/interaction for: *** *p* ≤ 0.001. According to Tukey’s HSD post hoc test, different letters indicate significant differences (*p* ≤ 0.05).
