# Supplementary material for: Sustainable Strategies to Counteract Mycotoxins Contamination and Cowpea Weevil in Chickpea Seeds during Post-Harvest
Source: Toxins (Basel). 2023 Jan 11;15(1):61. doi: 10.3390/toxins15010061 (PMC9865523; doi:10.3390/toxins15010061)
Supplement: Supplementary file 1 [file toxins-15-00061-s001.zip › toxins-2145218-supplementary.pdf]

toxins

Article

Sustainable Strategies to Counteract Mycotoxins Contamination and Cowpea Weevil in Chickpea Seeds during Post-harvest

Claudia Pisuttu, Samuele Risoli, Lorenzo Moncini, Cristina Nali, Elisa Pellegrini and Sabrina Sarrocco

Table S1. Analysis of variance of mycotoxins content in the four chickpea batches between the mean values determined on three different replicates.

Mycotoxin	Source of Variation	df	Mean square	F	*P*-value	F crit
Patulin	Between Groups	3	3919.22	4.616	0.0372	4.07
	Within Groups	8	849.07			
	Total	11				
AFG2	Between Groups	3	16.56	67.80	<0.001	4.07
	Within Groups	8	0.24			
	Total	11				
AFG1	Between Groups	3	0.02	24.79	<0.001	4.07
	Within Groups	8	0.00			
	Total	11				
AFB2	Between Groups	3	0.11	170.55	<0.001	4.07
	Within Groups	8	0.00			
	Total	11				
AFB1	Between Groups	3	0.01	145.56	<0.001	4.07
	Within Groups	8	0.00			
	Total	11				

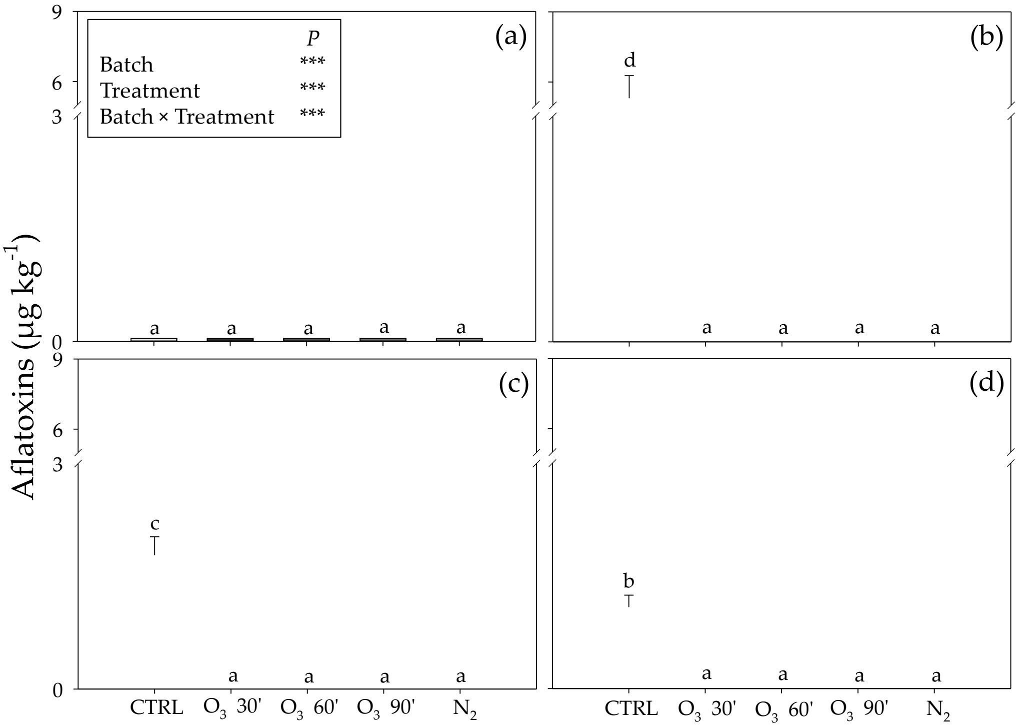

Figure S1. Total content of aflatoxins in four batches [n. 1 **(a)**, n. 2 **(b)**, n. 3 **(c)** and n. 4 **(d)**] of chickpea grains (CTRL, *white fill*), exposed to ozone [500 ppb O_3 for 30 (light grey fill), 60 (grey fill) and 90 (dark grey fill) minutes] and nitrogen treatment [99% N_2 for 21 consecutive days, dark fill]. Results of two-way ANOVA are reported, asterisks show the significance of factors/interaction for: *** $p \leq 0.001$. According to Tukey's HSD post hoc test, different letters indicate significant differences ($p \leq 0.05$).
